# Sub-inhibitory gentamicin pollution induces gentamicin resistance gene integration in class 1 integrons in the environment

**DOI:** 10.1038/s41598-023-35074-y

**Published:** 2023-05-27

**Authors:** Concepcion Sanchez-Cid, Timothy M. Ghaly, Michael R. Gillings, Timothy M. Vogel

**Affiliations:** 1 Environmental Microbial Genomics, UMR 5005 Laboratoire Ampère, CNRS, École Centrale de Lyon, Université de Lyon, Écully, France; 2 School of Natural Sciences, Macquarie University, NSW, 2109 Australia; 3 ARC Centre of Excellence in Synthetic Biology, Macquarie University, NSW, 2109 Australia; 4 Université de Lyon, Université Claude Bernard Lyon 1, UMR CNRS 5557, UMR INRAe 1418, VetAgro Sup, Ecologie Microbienne, F-69622 Villeurbanne, France

**Keywords:** Antimicrobial resistance, Bacterial genetics, Experimental evolution

## Abstract

Antibiotics at sub-inhibitory concentrations are often found in the environment. Here they could impose selective pressure on bacteria, leading to the selection and dissemination of antibiotic resistance, despite being under the inhibitory threshold. The goal of this study was to evaluate the effects of sub-inhibitory concentrations of gentamicin on environmental class 1 integron cassettes in natural river microbial communities. Gentamicin at sub-inhibitory concentrations promoted the integration and selection of gentamicin resistance genes (GmRG) in class 1 integrons after only a one-day exposure. Therefore, sub-inhibitory concentrations of gentamicin induced integron rearrangements, increasing the mobilization potential of gentamicin resistance genes and potentially increasing their dissemination in the environment. This study demonstrates the effects of antibiotics at sub-inhibitory concentrations in the environment and supports concerns about antibiotics as emerging pollutants.

## Introduction

The use of antibiotics to treat bacterial infections in humans and animals generates a flow of antibiotic residues into the environment^1,2^. Even treatment of wastewater cannot eliminate antibiotics, and residual concentrations are released into the environment^3,4^. Consequently, antibiotics can be found in environmental settings, although usually at relatively low concentrations^5^. These concentrations are often considered to be sub-inhibitory, being too low to induce a significant inhibition of bacterial growth^6^. However, sub-inhibitory concentrations of antibiotics can trigger the SOS response^7^, select for antibiotic resistance^8,9^ and stimulate horizontal gene transfer^10–12^ in vitro. Therefore, sub-inhibitory concentrations of antibiotics might induce the selection and dissemination of antibiotic resistance in the environment^13,14^ and promote the dissemination of environmental resistance genes to human pathogens^15^.

The term “sub-inhibitory”, which was originally described for pure cultures, is often applied to complex communities^16–20^. However, the members of a complex community differ in their response to antibiotics, with some members being inhibited and some members benefiting from the same antibiotic concentration^21^. Therefore, there is a rising concern that environmentally relevant antibiotic concentrations (which are often described as sub-inhibitory and do not limit overall community growth) can select for antibiotic resistance in the environment^21^. In addition, they could increase the dissemination of ARGs in the environment and to human bacteria. Since these putative sub-inhibitory concentrations kill fewer members of the bacterial community than inhibitory concentrations and it has been suggested that antibiotics at sub-inhibitory concentrations could have a signaling role in complex communities^5,22^, these low concentrations might create a risk for human health by triggering responses in environmental bacteria that lead to efficient dissemination of antibiotic resistance genes (ARGs) to a wider host range than inhibitory concentrations.

Class 1 integrons are key genetic elements in the dissemination of antibiotic resistance^23,24^ and they are often used as proxies for anthropogenic pollution^25^. They can reversibly integrate antibiotic resistance genes in their cassette arrays via the class 1 integrase, IntI1^26^. The gene cassettes integrated in class 1 integron arrays can subsequently be expressed from a promoter, Pc, located at the start of the array^27^. Therefore, gene cassettes located closer to the promoter benefit from a higher expression. In addition, class 1 integrase activity is regulated by the SOS response^28,29^ . Antibiotics that induce the SOS response at sub-inhibitory concentrations induced integrase expression in *E. coli*^30^*.* Therefore, the exposure of environmental bacteria to sub-inhibitory concentrations of antibiotics might induce structural changes in class 1 integrons and have an impact on the mobilization potential of antibiotic resistance genes. The goal of this study was to determine whether gentamicin at sub-inhibitory concentrations (as determined by growth inhibition in vitro and confirmed by a 16S rRNA gene qPCR in microcosms)^21^ affects the structure of class 1 integrons. We hypothesized that gentamicin at sub-inhibitory concentrations could: (a) induce the integration of GmRG in class 1 integrons by increasing the activity of the class 1 integrase, (b) select for class 1 integron cassettes that already contained GmRG at the beginning of the experiment and/or (c) induce a rearrangement of GmRG in class 1 integron cassettes to positions closer to the promoter so that they are more readily transcribed. To the best of our knowledge, this is the first study demonstrating that sub-inhibitory concentrations of antibiotics can induce changes in class 1 integrons in the environment, thus increasing the potential for mobilization and subsequent dissemination of ARGs.

## Materials and methods

### qPCR of the class 1 integrase intI1 gene from river water

Details of sampling and microcosm set-up can be found in Sanchez-Cid et al.^21^*.* Briefly, 1L Rhône river water microcosms were exposed to sub-inhibitory (10 and 50 ng/ml) and inhibitory (800 ng/ml) concentrations of gentamicin for two days, as well as from non-polluted controls. Triplicates were made for each antibiotic concentration. DNA was extracted after 0, 1 and 2 days. In this study, the class 1 integrase gene (*intI1*) was amplified using HS463a (5′-CTGGATTTCGATCACGGCACG-3′) and HS464 (5′-ACATGCGTGTAAAT-CATCGTCG-3′) primers^31^. Quantitative PCR assays were done using the Corbett Rotor-Gene 6000 (QIAGEN, Hilden, Germany) in a 20 µl volume containing GoTaq PCR Master Mix (Promega), 0.75 µM of each primer and 2 µl of DNA. Two non-template controls were also included in the assay. The standard sample was obtained from water DNA and cloned and transformed using the TOPO TA cloning Kit (Thermo Fisher Scientific). The standard was normalized to 10^8^ copies/µl and standard curves were made in triplicate using tenfold serial dilutions (10^7^–10^2^ per µl of qPCR reaction). Amplification conditions were 95 °C for 2 min followed by 35 cycles of 95 °C for 15 s, 60 °C for 30 s and 72 °C for 30 s. Melting curves were generated by increasing the temperature from 60 to 95 °C. Primer efficiency was 1.02 and the linearity R^2^ coefficient was 0.98. The number of copies of *intI1* were normalized by the number of copies of the 16S rRNA gene^21^ to determine the relative abundance of the class 1 integrase gene in water microcosms (Figure S1 in Supplementary Information). Statistical differences between the conditions (gentamicin concentration and exposure time) were evaluated using the Kruskal–Wallis test.

### Long-read sequencing of class 1 integron cassettes from river water

Class 1 integron cassettes had been previously amplified from river water DNA using MRG284 (5’- GTTACGCCGTGGGTCGATG-3’) and MRG285 (5′- CCAGAGCAGCCGTAGAGC-3′) primers^21^. Triplicates from each condition (gentamicin concentration and exposure time) were pooled prior sequencing to ensure sufficient input. Libraries were prepared from the 12 resulting samples using the Ligation Sequencing Kit SQK-LSK109 and the Native Barcoding Expansion 1–12 (Oxford Nanopore) according to the “Native Barcoding protocol” described by Oxford Nanopore. The normalized and pooled library was sequenced using a FLO-MIN106 (R9.4.1) flow cell. Sequencing depth obtained from each triplicate pool can be found in Table S1 in Supplementary Information. Sequences were basecalled using Guppy basecaller v6.0.1 high accuracy model (Oxford Nanopore). The percentage of GmRG in the reads was determined by blasting the sequences against the CARD database^32^ (Figure S2 in Supplementary Information). Long-read integron amplicon sequences were first oriented and trimmed based on the forward and reverse primer sequences (MRG284/285) using Pychopper v2.6.0 (https://github.com/epi2me-labs/pychopper) [paramters: -m edlib]. Reads that did not contain both primers in the correct arrangement were discarded. The primer-oriented reads were then clustered into amplicon-specific clusters using isONclust v0.0.6.1^33^ [parameters: –ont –fastq | write_fastq –N 1]. Error correction was then performed on each cluster using isONcorrect v.0.0.8^34^ with default parameters, and a single consensus sequence was generated for each cluster using spoa v4.0.7^35^ [parameters: -r 0]. Finally, annotation of gene cassettes and *attC* recombination sites for each consensus sequence was performed using IntegronFinder v2.0rc6^36^ [parameters: –local-max –gbk –calin-threshold 1].

### Second sequencing of samples exposed to gentamicin at 50 and 800 ng/ml of gentamicin at day 0

Pooled triplicates exposed to gentamicin at 50 ng/ml and at 800 ng/ml at day 0 were resequenced to increase sequencing depth. Two sequencing runs were performed (one per each triplicate pool) using the Ligation Sequencing Kit SQK-LSK109 and FLO-MIN106 (R9.4.1) flow cells. Sequencing depth obtained from each triplicate pool can be found in Table S1 in Supplementary Information. Sequences were base called using the Guppy basecaller (Oxford Nanopore). Then, the sequences were blasted against consensus integron cassette arrays of interest identified in the first sequencing run in order to determine whether these cassettes were present in water microcosms at the beginning of the experiment.

### Informed consent

Correspondence and material requests should be addressed to Concepcion Sanchez-Cid: concepcion. sanchezcid-torres@ec-lyon.fr.

## Results

**Figure 1:**
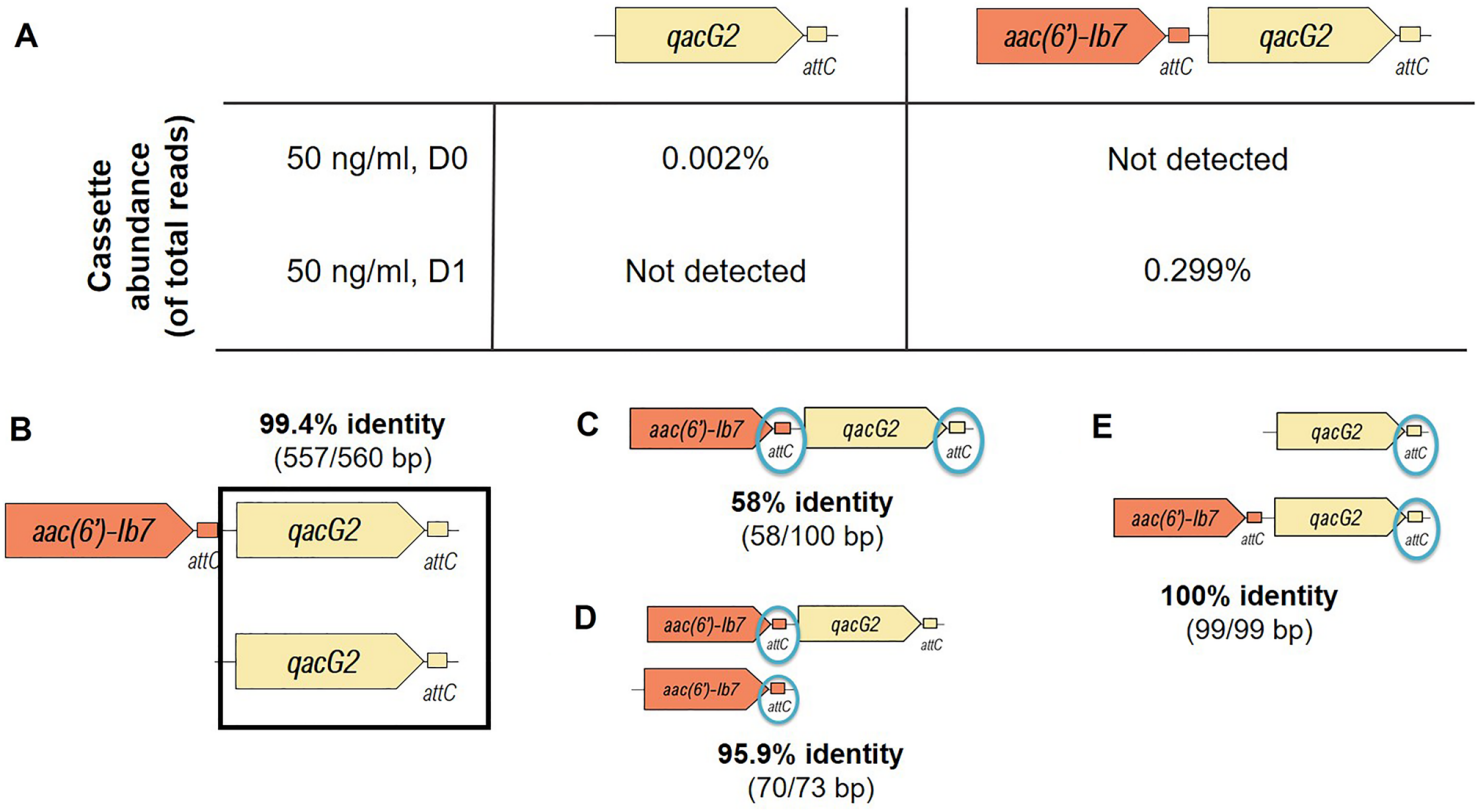
Gentamicin resistance gene (GmRG) integration event induced by gentamicin at sub-inhibitory concentrations (50 ng/ml). (**A**) Abundance (% of total reads) at day 0 and day 1 of the precursor class 1 integron cassette containing a *qacG2* gene and of the cassette with an integrated GmRG *aac(6’)-Ib7*. (**B**) Identity between *qacG2* gene and its associated *attC* site in the precursor cassette and the cassette with the integrated GmRG. (**C**) Identity between the *attC* site associated to *aac(6’)-Ib7* and the one associated to *qacG2*. (**D**) Identity between two *attC* sites associated to the *aac(6’)-Ib7* gene in two different cassettes. (**E**) Identity between the *attC* sites associated to the *qacG2* gene in the precursor class 1 integron cassette containing a *qacG2* gene and of the cassette with an integrated GmRG *aac(6’)-Ib7*.

In water microcosms exposed to 50 ng/ml of gentamicin (a sub-inhibitory concentration), a 1239 bp long cassette array containing a GmRG (99.5% of identity to *aac(6’)-Ib7)* and a quaternary ammonium compound resistance gene, *qacG2,* was identified in the sequence run after 1 day exposure (Fig. 1A). This cassette had 578 reads at day 1 and was not detected in the same microcosms at day 0. On the other hand, the potential ancestor of the array, which contained only the *qacG2* gene and had a length of 600 bp, was observed at day 0 (17,790 reads) but not at day 1 (Fig. 1A). The sequence containing the *qacG2* gene and its associated *attC* recombination site was almost identical between the two cassettes (99.4% identity) except for 3 nucleotides (Fig. 1B). The identity between the *attC* recombination site associated to the GmRG *aac(6’)-Ib7* and the one associated with the *qacG2* gene in the cassette detected at day 1 was 58% (Fig. 1C), whereas the identity between two *attC* sites associated to the same gene (*aac(6’)-Ib7)* in two different class 1 integron cassettes was 95.9% (Fig. 1D). However, only the two *attC* sites associated with the *qacG2* gene in the two cassettes shown in Fig. 1A (i.e*.,* the potential ancestor cassette and the cassette containing a GmRG and a *qacG2* gene) showed 100% identity (Fig. 1E).

**Figure 2:**
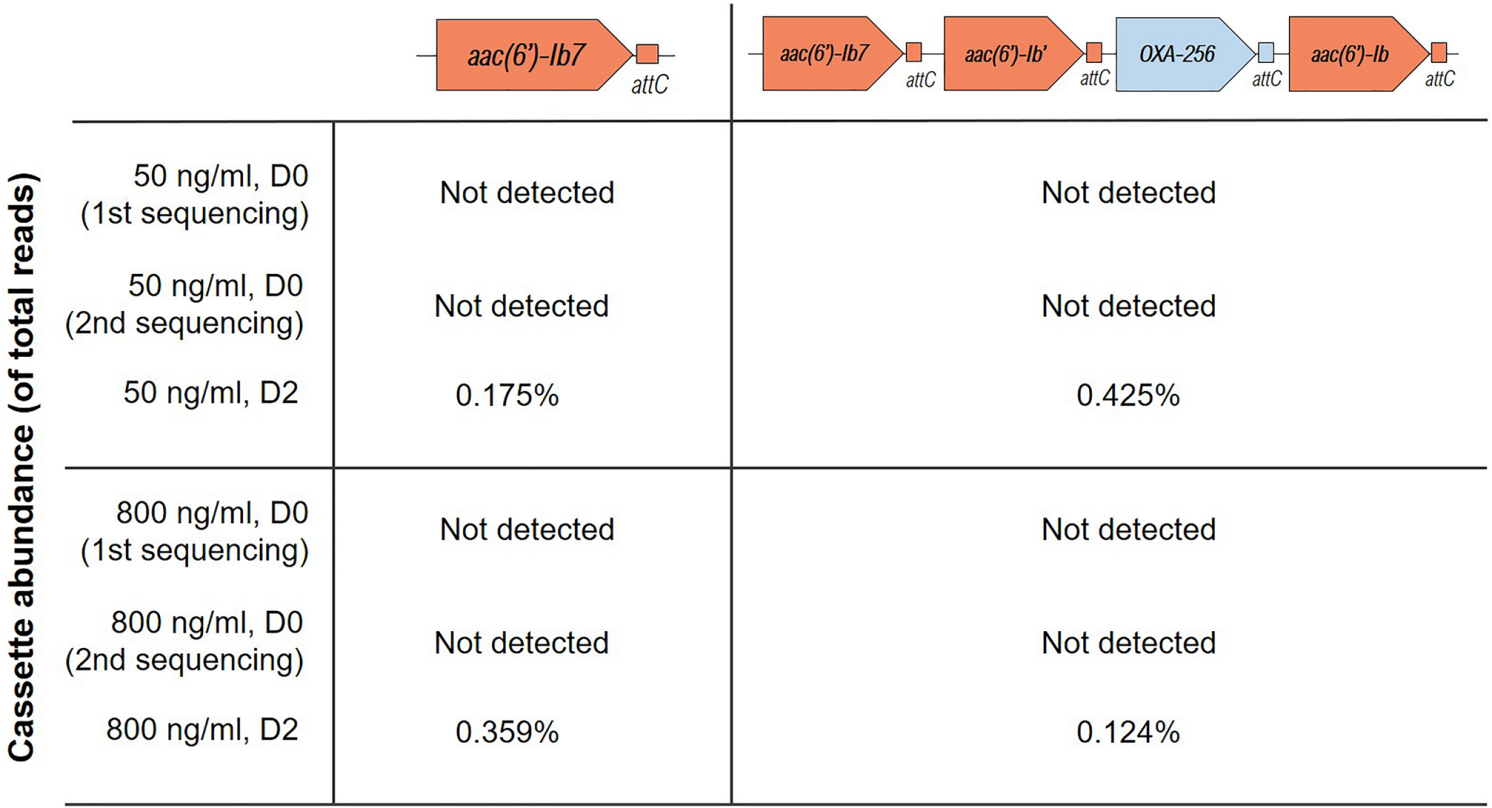
Abundance (% of total reads) of two cassette arrays containing gentamicin resistance genes in water microcosms exposed to 50 ng/ml (sub-inhibitory) and 800 ng/ml (inhibitory) of gentamicin after 0 and 2-day exposure. Samples at day 0 were resequenced to increase sequencing depth.

In addition, two cassette arrays carrying GmRG were found in water microcosms exposed to both 50 ng/ml (sub-inhibitory) and 800 ng/ml (inhibitory) of gentamicin after a 2-day exposure (Fig. 2). The first contained one GmRG, the *aac(6’)-Ib7* gene (99.6% identity)*.* This cassette with a single GmRG was more abundant in the nanopore reads at inhibitory concentrations with 392 reads at 50 ng/ml and 1151 reads at 800 ng/ml of gentamicin. The second array was 2930 bp long and contained three GmRG (one *aac(6’)-Ib7*; 100% identity and two *aac(6’)-Ib* genes; 99.6% and 99.5% identity respectively) and a beta-lactamase gene, *OXA-256.* This cassette was more abundant at sub-inhibitory concentrations than at inhibitory concentrations with 953 reads at 50 ng/ml and 399 at 800 ng/ml of gentamicin. Neither of the two cassettes were detected at the beginning of the experiment even after day 0 samples were resequenced to increase the sequencing depth ~ 38-fold increase for samples exposed to 50 ng/ml of gentamicin and ~ 23-fold increase for samples exposed to 800 ng/ml (Table S1 Supplementary Information). Other ARGs, *aadA* aminoglycoside resistance genes and beta-lactamases, were detected in all samples (Table S2 in Supplementary Information). Whereas no apparent effect of gentamicin pollution on the presence of the widely distributed *aadA* gene in class 1 integrons was detected, a higher percentage of integrons contained beta-lactamases after exposure to gentamicin at 50 and 800 ng/ml for two days. In samples exposed to 10 ng/ml of gentamicin (sub-inhibitory) and non-polluted controls, no observable effects were detected in the structure of class 1 integrons.

## Discussion

Sub-inhibitory concentrations of antibiotics could have ecological effects on environmental bacteria^37,38^ and lead to an increased selection and dissemination of ARGs in the environment^21,39^. This could result in the acquisition of new resistance mechanisms by human microbiome bacteria and aggravate the worldwide antibiotic crisis^40,41^. The goal of this study was to determine whether gentamicin at overall sub-inhibitory concentrations induces structural changes in class 1 integrons, which are widely distributed in the environment and play a major role in antibiotic resistance dispersal^42,43^. Our results demonstrate an effect that sub-inhibitory concentrations of antibiotics could have in the environment by observing that gentamicin at sub-inhibitory concentrations was associated with altered structures of class 1 integrons in environmental samples.

Even though some variability was observed between triplicates, gentamicin at sub-inhibitory concentrations did not alter overall class 1 integron abundance (Figure S1 in Supplementary Information), which only increased under (growth) inhibitory conditions. However, the increase in the proportion of GmRG found in class 1 integron cassettes (Figure S2 in Supplementary Information) is consistent with a specific selective response to gentamicin at sub-inhibitory concentrations. Moreover, whereas no structural changes in class 1 integrons were measurable at 0 and 10 ng/ml of gentamicin, some effects were observed in river water exposed to 50 ng/ml of gentamicin, a sub-inhibitory concentration, and to 800 ng/ml, an inhibitory concentration. Thus, although some sub-inhibitory concentrations of antibiotics (i.e.*,* 10 ng/ml) might be too low to have a selective potential on environmental class 1 integrons, the selective threshold is much lower than the inhibitory threshold^44^ and sub-inhibitory antibiotic pressure could contribute to the evolution of these genetic elements.

Our research results are consistent with gentamicin at 50 ng/ml inducing the integration of GmRG in class 1 integrons. We used the sequence analysis of the *attC* recombination sites^45^ (Fig. 1) to identify an integration event and the precursor cassette in microcosms polluted at this sub-inhibitory concentration. Given the high sequence variability of *attC* sites^46^, the 100% sequence identity of the *attC* recombination sites associated with the *qacG2* gene in the cassette with a GmRG and its precursor (Fig. 1E) supports the hypothesis that gentamicin sub-inhibitory pressure induced the integration of a GmRG in a pre-existent cassette array containing a *qacG2* gene. In addition, the detection of two identical GmRG-containing class 1 integron cassettes under sub-inhibitory and inhibitory pollution after 2-days exposure might indicate that some of the processes induced by gentamicin at inhibitory concentrations can also be induced by sub-inhibitory concentrations of gentamicin (or vice versa). Although some structural changes were observed both at sub-inhibitory and inhibitory concentrations, an integration event was detected only at sub-inhibitory concentrations. Thus, these low concentrations of antibiotics could have a greater impact on class 1 integron evolution than inhibitory concentrations. These observations underline the concern that sub-inhibitory concentrations of antibiotics can pose a risk for human health^13^ and they could arguably be more dangerous than inhibitory concentrations^47,48^, since they can exert a selective pressure with less lethal effects on target bacteria. Therefore, future research should be focused on the impacts of antibiotics on antibiotic resistance selection in all the different members of the community, regardless of their effects on growth inhibition. The lack of detection of these cassettes (Fig. 2) and of GmRG and the *OXA-256* gene (Table S3 in Supplementary Information) at the beginning of the experiment, even at relatively high sequencing depths, supports the integration of these genes into previously empty class 1 integron arrays as the mechanism driving the presence of these cassettes at day 2 under both sub-inhibitory and inhibitory conditions. This mechanism is also supported by the increase in the proportion of beta-lactamase-containing class 1 integrons under gentamicin pollution (Table S2 in Supplementary Information). Although the original genomic contexts of these GmRG cassettes is unknown, their absence from class 1 integrons at day 0 is consistent with class 1 integrons capturing cassettes that confer strong selective advantages from (non-class 1) chromosomal integrons, and thus drastically increasing the mobilization potential of GmRG.

Although our results only support the hypothesis that gentamicin at sub-inhibitory concentrations induced the integration of GmRG in class 1 integron cassettes, the other two mechanisms that we hypothesized could be induced by sub-inhibitory pressure (i.e*.,* selection of preexistent GmRG-containing integron cassettes and rearrangement of GmRG to positions closer to the promoter) cannot be overruled. We did not observe any GmRG-containing class 1 integron cassettes at the beginning of the experiment (Table S3 in Supplementary Information) that could be selected under gentamicin pressure. On the other hand, the class 1 integrase mediates not only the integration of genes in class 1 integron cassettes but also gene rearrangement within the cassette arrays^49^. Therefore, this mechanism could also be induced by sub-inhibitory pollution, although we have found no evidence to support this hypothesis here.

Since the activity of the class 1 integrase, IntI1, is regulated by the SOS response, the increase of integration of GmRG in class 1 integron cassettes observed at both sub-inhibitory and inhibitory concentrations could be SOS-mediated. Previous research suggested that sub-inhibitory concentrations of aminoglycosides induce integrase activity in *E. coli* via the SOS response^50^. As long as an antibiotic is present at a concentration high enough to induce a selective pressure on one member of the bacterial community, the SOS response could be activated in response to the DNA damage induced by antibiotic pollution. This activation could lead to an integration of resistance genes into class 1 integron cassette arrays, regardless of the potential of that antibiotic concentration to induce overall growth inhibition. However, we were not able to test this hypothesis in this study and further research should explore this question.

In conclusion, this study demonstrated that gentamicin at sub-inhibitory concentrations can have an impact on the structure and evolution of class 1 integrons in the environment and increase the mobilization potential of GmRG. Subsequently, the genes that were recruited into class 1 integrons under sub-inhibitory antibiotic pressure could be further disseminated between environmental bacteria and potentially to human microbiome bacteria, including human pathogens^51,52^. In this study we have not addressed the dissemination potential of these integron cassettes in the environment and their potential recruitment by human microbiome bacteria. Further studies should evaluate the risks associated to these possibilities, both in the presence and absence of selective sub-inhibitory pressure.

## Supplementary Information

Supplementary Information 1.

## Data Availability

The datasets generated and analyzed for this study are publicly available in the Genome Sequence Archive (BioProject PRJCA013931, accession number CRA009410).
