# Supplementary material for: Sub-inhibitory gentamicin pollution induces gentamicin resistance gene integration in class 1 integrons in the environment
Source: Sci Rep. 2023 May 27;13:8612. doi: 10.1038/s41598-023-35074-y (PMC10224954; doi:10.1038/s41598-023-35074-y)
Supplement: Supplementary file 1 — Supplementary Information 1. [file 41598_2023_35074_MOESM1_ESM.pdf]

Sub-inhibitory gentamicin pollution induces gentamicin resistance gene integration in class 1 integrons in the environment

Concepcion Sanchez-Cid[1*], Timothy M. Ghaly[2], Michael R. Gillings[2,3], Timothy M. Vogel[4]

[1]Environmental Microbial Genomics, CNRS UMR 5005 Laboratoire Ampère, École Centrale de Lyon, Université de Lyon, Écully, France

[2]School of Natural Sciences, Macquarie University, NSW, 2109, Australia

[3]ARC Centre of Excellence in Synthetic Biology, Macquarie University, NSW, 2109, Australia

[4]Université Claude Bernard Lyon 1, CNRS, INRAE, VetAgro Sup, UMR Ecologie Microbienne, Villeurbanne, F-7 69622 France

Supplementary Information

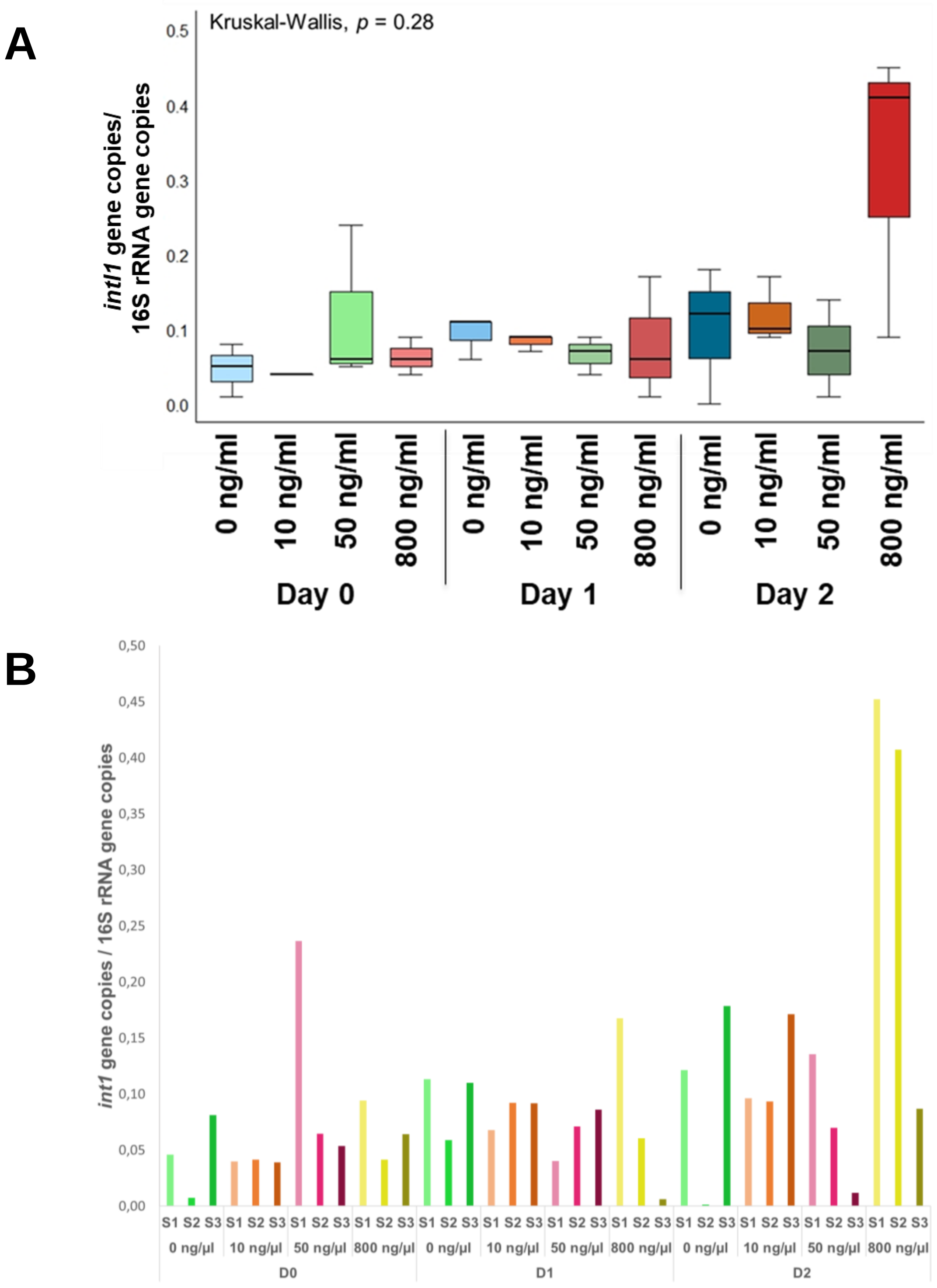

Figure S1. Relative abundance of the *intI1* in river water microcosms exposed to different gentamicin concentrations over 2 days. A) Average relative abundance. B) relative abundance of individual triplicates (green: 0 ng/ml, orange: 10 ng/ml, pink: 50 ng/ml, yellow: 800 ng/ml). Relative abundance was normalized by the number of copies of the 16S rRNA gene. n=3.

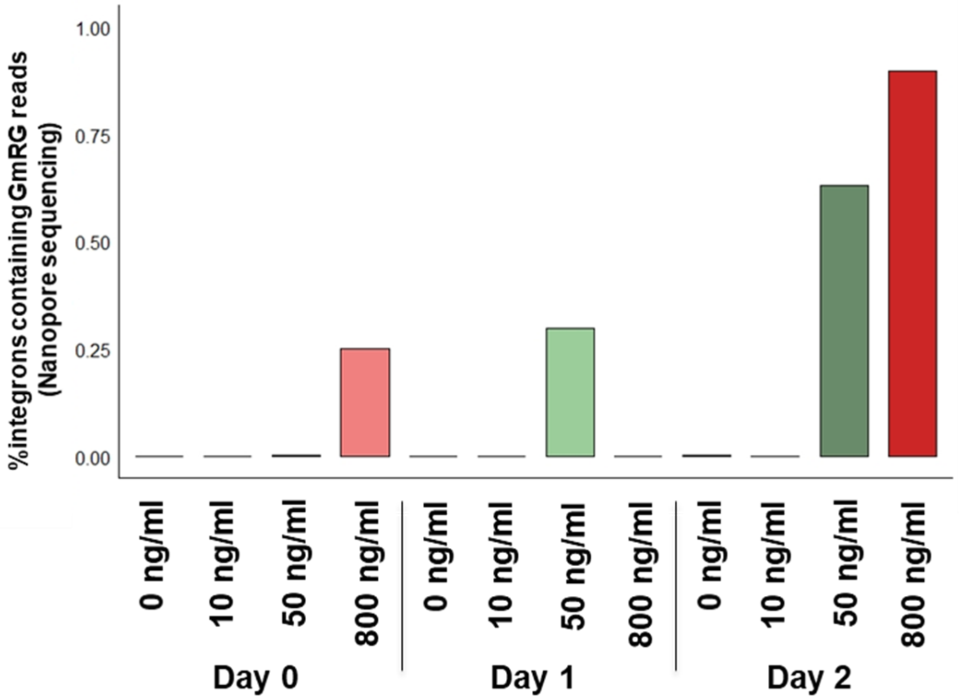

Figure S2. Percentage of integrons containing GmRG from total sequences obtained from river water microcosms exposed to different gentamicin concentrations over 2 days. Triplicates were pooled prior sequencing to ensure sufficient input.

Exposure time	Gentamicin concentration	Sequencing depth (first sequencing)	Sequencing depth (second sequencing)
**Day 0**	**0 ng/ml**	457590	NA
	**10 ng/ml**	672553	NA
	**50 ng/ml**	231735	9006578 (~38-Fold increase)
	**800 ng/ml**	362271	8488398 (~23-Fold increase)
**Day 1**	**0 ng/ml**	69957	NA
	**10 ng/ml**	221514	NA
	**50 ng/ml**	193401	NA
	**800 ng/ml**	223192	NA
**Day 2**	**0 ng/ml**	483927	NA
	**10 ng/ml**	254607	NA
	**50 ng/ml**	224073	NA
	**800 ng/ml**	320832	NA

Table S1. Sequencing depth obtained from the long-read sequencing of pooled triplicates. NA = not resequenced samples.

Exposure time	Gentamicin concentration	Number of unique class 1 integron cassettes	Percentage of integrons containing *aadA* genes	Percentage of integrons containing beta-lactamases
**Day 0**	**0 ng/ml**	49	0.77	0.10
	**10 ng/ml**	50	3.81	0.01
	**50 ng/ml**	49	4.84	0.06
	**800 ng/ml**	67	5.58	0.31
**Day 1**	**0 ng/ml**	35	0.54	0.06
	**10 ng/ml**	68	9.06	0.04
	**50 ng/ml**	59	2.21	0.08
	**800 ng/ml**	43	4.49	0.02
**Day 2**	**0 ng/ml**	107	5.43	0.04
	**10 ng/ml**	91	4.90	0.06
	**50 ng/ml**	67	5.75	16.57
	**800 ng/ml**	50	1.23	16.65

Table S2. Number of unique class 1 integron consensus cassettes and percentage of integrons containing other antibiotic resistance genes from total sequences obtained from river water microcosms exposed to different gentamicin concentrations over 2 days. Antibiotic resistance genes were identified by blasting the sequences against the CARD database.

	GmRG	OXA-256
**GM 50 ng/ml, Day 0 (2nd sequencing)**	-	-
**GM 800 ng/ml, Day 0 (2nd sequencing)**	-	-
**GM 50 ng/ml, Day 2 (positive control)**	+	+
**GM 800 ng/ml, Day 2 (positive control)**	+	+

Table S3. Presence/absence of GmRG and the beta-lactamase *OXA-256* in the reads obtained from the second sequencing of class 1 integrons from water microcosms exposed to 50 ng/ml (sub-inhibitory) and 800 ng/ml (inhibitory) of gentamicin after 0 and 2-day exposure. GmRG and the *OXA-256* gene were identified by blasting the sequences against the CARD database.
